# Exploring the Experiences of Times Without Care and Encounters in Persons With Dementia in the Swiss and German Nursing Home and Domiciliary Care Settings: Protocol for an Ethnographic Multimethods Study

**DOI:** 10.2196/58190

**Published:** 2024-11-18

**Authors:** Thomas Beer, Julian Hirt, Laura Adlbrecht, Ulrike Lindwedel, Matthias Dammert, Carola Maurer, Matthias Kliegel, Peter König, Helma M Bleses

**Affiliations:** 1 Department of Health, Eastern Switzerland University of Applied Sciences St.Gallen Switzerland; 2 Pragmatic Evidence Lab, Research Center for Clinical Neuroimmunology and Neuroscience Basel (RC2NB), University Hospital Basel and University of Basel Basel Switzerland; 3 Institute of Health and Nursing Science, Medical Faculty, Martin Luther University Halle-Wittenberg Halle (Saale) Germany; 4 Faculty Health, Safety, Society, Furtwangen University Furtwangen Germany; 5 Department of Health Science, Fulda University of Applied Sciences Fulda Germany; 6 Center for the Interdisciplinary Study of Gerontology and Vulnerability (CIGEV), University of Geneva Geneva Switzerland; 7 Department of Psychology, University of Geneva Geneva Switzerland; 8 Swiss National Center of Competences in Research LIVES–Overcoming vulnerability, Life-Course Perspectives Lausanne and Geneva Switzerland

**Keywords:** dementia, nursing homes, home nursing, home care services, nursing, ethnography, formal caregivers, informal caregivers

## Abstract

**Background:**

Persons with dementia spend a large part of the day without care and encounters, often without activity, as confirmed by numerous studies. However, no scientific analysis has examined how persons with dementia experience these periods. Such knowledge would be highly relevant for health care professionals and relatives to develop adequate strategies for dealing with times without care.

**Objective:**

We aim to reconstruct times without care and encounters in persons with dementia in the nursing home and domiciliary care settings and develop a typology. This typology will address the lifeworld understandings of time and the ways of arranging the time of persons with dementia.

**Methods:**

Our study is designed as an explorative, sequential multimethods investigation. We aim to systematically reconstruct times without care and encounters over a period of 36 months using ethnographic methods. Afterward, we will examine the resulting typology using a survey. To describe different social and caring cultures, practices, and arrangements, we will analyze time periods across all phases of dementia in (1) institutions exclusively caring for persons with dementia, (2) institutions where persons with dementia and those without live together, and (3) domiciliary care. For each type of care, our target is 10 intensive case observations. These observations will occur in both *participatory* and *nonparticipatory* ways. We video record selected situations and conduct situational conversations and interviews with persons with dementia and nurses. We are aiming for a minimum sample of 30 persons with dementia plus their caregivers (ie, relatives of people with dementia and professional caregivers). We will analyze data according to grounded theory methodology. Furthermore, we will perform a hermeneutic sequence analysis of selected text passages. To interpret the video material, we will conduct a video interaction analysis. To obtain complementary information about the newly developed typology, we will survey approximately 400 formal and 150 informal caregivers. We will summarize the ethnography and survey findings into an overall concept of times without care and encounters in persons with dementia. To fulfill the research objectives, our cross-disciplinary and cross-country team comprises researchers with expertise in nursing sciences, gerontology, sociology, psychology, and ethnography.

**Results:**

Our approach allows formulating statements about the nature, frequency, and prevalence of times without care and encounters in people with dementia across countries and types of care. Thus, we will contribute to making visible the lifeworld of persons with dementia. Our study commenced in March 2022 and will conclude in May 2025. The results are expected to be published in the fall of 2025.

**Conclusions:**

Our research offers points of departure for the representative investigation of times without care and encounters in persons with dementia, for the development of diagnostic instruments, and for dealing critically with possibilities of interruption (eg, by developing targeted interventions).

**International Registered Report Identifier (IRRID):**

DERR1-10.2196/58190

## Introduction

### Background

The diagnosis and progression of dementia are associated with lifeworld crises because the patterns of interpretation and action increasingly fail, and there is uncertainty and a lack of orientation [1,2]. Such crises arise and persist in the working and care worlds located in domestic-familial and institutional settings. For the most part, these crises receive political and social acceptance. Isolation, loneliness, helplessness, excessive demands, and despair, as well as the associated severe restrictions on basic rights and liberty, are characteristic of the lifeworld contexts experienced by persons with dementia and their caregivers [3-6].

Persons with dementia and their caregivers *navigate* a marginalized world characterized by the collapse of the structures of help, care, support, and relationships. The associated ethical conflicts and dilemmas are politicized and discussed in society. However, this does not change the affected persons’ experience of crisis [7-12].

The issue of dementia has played an enormous role in the political, social, and scientific debate for several years. This is now reflected in the German-speaking population. As the German Dementia Barometer 2018 shows, the majority of the population feels well informed about dementia and believes that there are good opportunities for supporting persons with dementia [13].

However, at the same time, a marginalization of the dementia phenomenon is evident at various levels. The review by Lara et al [14] indicates the interrelation between subjectively experienced loneliness and the risk of developing dementia. According to the findings, people with a subjective feeling of marginalization are particularly affected by dementia. They feel socially excluded even before they are diagnosed. By contrast, the pathologization or stigmatization of persons with dementia as people that suffer from dementia increases their marginalization. Due to their varying degrees of loss of cognitive and communicative abilities, persons with dementia are less and less involved in social life in the sense of social participation. Caring for persons with dementia increasingly takes place in a social protective sphere [15] (eg, in a familial, dyadic care dependency or in a care facility). The social, restrictive area of protection results predominantly from a caring motivation or a benevolent compulsion” [16]. Country-specific differences could play a role here: in Switzerland, caring for and supporting persons with dementia in the institutional setting predominantly occurs in so-called protected and secured living areas [17,18]. Unlike in Germany, these protected living areas are usually isolated from other normal living areas. As a result, persons with dementia become even more fixed in place, immobile, and therefore isolated agents at the margin [19-21]. Persons with dementia enter a downward spiral due to inadequate cognitive, emotional, and sensory stimulation. This has a negative impact—on the course of dementia; the development of challenging behavior; the way of shaping relationships with their environment; and, above all, their physical, cognitive, and emotional states [22]. Almost on the basis of a conceptual justification and thus in a socially legitimized way, persons with dementia are isolated and immobilized [21,23]. This imposed solitude seems to support the *production* and *potentiation* of care dependency in persons with dementia [24,25]. The scientific and social discourse on this closed-door policy takes place internationally [17,26-29].

There are numerous empirical indications that persons with dementia in institutional care settings experience long periods of boredom and inactivity, along with associated phases of loneliness [21,30-36]. In addition, observational studies document the lack of activation of thoughts or behaviors and involvement of persons with dementia. Accordingly, during the majority of the observation periods, persons with dementia were found sitting alone, being inactive, resting, dozing, sleeping, or sitting or lying in front of the television set [35,37-39]. Persons with dementia spend an average of 5 hours a day asleep [40].

It should be noted that activity programs in inpatient care settings only take place to a limited extent [41]. Theurer et al [33] confirm institutionalized inactivity in their study on physical activity in institutional long-term care. Two observational studies [42,43] examined the activities of persons with dementia during the day in different long-term care settings. Both studies found significantly more activity and social interaction in specialized institutions. However, also in these settings, persons with dementia seem to spend a relatively large amount of time in nonpurposeful activity (eg, tapping on a table, rubbing hands for no reason, picking at clothing or objects, wandering, and mumbling) or inactivity. These findings are based exclusively on quantitative observational data and represent the researchers’ perspective. As a result, it was not possible to draw conclusions about how people with dementia themselves perceive these periods of time. Therefore, there may be a difference in perspective [44].

In a Swedish ethnographic study addressing the situation in nursing homes, Harnett [45] described these periods of time as “respite spaces.” A large part of the life of persons with dementia seems to consist of respite spaces. During these periods of time, persons with dementia neither communicate nor interact with nurses. They are not involved in interventions or routine processes. Harnett [45] advocated intensive investigation of these respite spaces to understand what they mean for people with dementia and their caregivers. Harnett [45] observed that persons with dementia not only passively submit to institutional events but also actively shape them—by using communicative strategies and entering into contact with each other. In respite spaces, persons with dementia seem to construct an intersubjectively shared reality of life. However, they usually do not organize their own daily routines—they see nurses as responsible for the social organization of a living unit [45]. In community rooms, they try to fulfill the shared expectation of sitting still and taking care of their own things [46]. By contrast, nurses’ practices seem to be directed at *prompting* persons with dementia to sit as still as possible and mind their own business [46,47]. Other reasons for the low level of activity and involvement may be physical impairments, obstructive architecture (eg, large, noisy community rooms), and unfavorable design of the living environment. Limited time resources or a shortage of (nursing) staff might also play a role [48,49]. Together, these factors limit opportunities and choices to participate in activities and social relationships that persons with dementia consider important [50-52].

According to an explorative, Scandinavian study based on qualitative interviews with persons with dementia and nurses, persons with dementia wish to be more involved in activities and relationships. This is important for them to lead a dignified life [53]. By contrast, nurses often consider persons with dementia as inactive and unmotivated [51]. For their part, persons with dementia seek to engage in individual and group activities. These activities are important to them [53] and help them to live a meaningful and fulfilling life [54]. In addition, persons with dementia can experience and coconstruct their identity in activities that are meaningful to them [55]. Relationships characterized by respect and goodwill, in which they feel heard, understood, and accepted, are important for them [56,57]. In the domiciliary care setting, family caregivers also emphasize the importance of routines and sense-giving activities to maintain the continuity and stability of the care situation [58].

In times without care and encounters, persons with dementia may strive for activities that resemble to those generally described as leisure activities. Studies on the importance of leisure for persons with dementia are congruent with the sociopsychological concept of leisure [59]. According to Prahl [60], leisure has become a dominant area of modern societies. It refers to people’s *free* time [60]. Leisure varies across social groups in terms of quantity and quality. For persons with dementia, access to leisure activities understood in this way is often difficult. They experience leisure time in increasing dependence on family members or other people. As a result, the leisure time of persons with dementia (as well as that of their informal caregivers) is condensed in terms of time and space.

However, engaging in or participating in activities considered leisure activities can have a positive impact on the well-being of persons with dementia and their families [59]. Leisure activities can be an opportunity for self-fulfillment. They allow persons with dementia to “be me, be with, make a difference, seek freedom, find balance, grow and develop and have fun” [61]. In relation to persons with advanced dementia, the literature does not generally refer to leisure activities but to meaningful activities. However, for the most part, meaningful activities coincide with leisure activities, as shown by a synthesis of qualitative studies on the perspective of persons with dementia regarding meaningful activities [62]. In a British grounded theory study, family caregivers emphasize the need to constantly reassess and adapt the goals, content, and types of activities that are meaningful for persons with dementia [58].

The authors of a meta-ethnography on the meaningful activities of persons with dementia underline the relevance of meaningful activities for a fulfilling life for persons with dementia [54]. They point out that these activities evolve and change with a person’s identity [54]. Supporting meaningful activities for persons with dementia seems to be challenging for nurses. Nurses consider those activities as essential that reflect the interests of the person with dementia; activities should be personalized and fit into the social or physical environment. However, they face certain barriers preventing the introduction and implementation of meaningful activities: structural (barriers related to maintaining organizational routines), personal (barriers arising from insufficient specific knowledge of activities, roles, and habits before taking up employment in a nursing home), and interprofessional (barriers stemming from a lack of awareness of meaningful activities among professionals). Nurses also identify a low awareness of meaningful activities [63,64].

Times without care and encounters can also have a negative impact. Low levels of physical or cognitive activity may have a long-term influence on the progression of cognitive impairment and mortality in persons with dementia [65,66]. During periods of low social involvement, Cohen-Mansfield et al [23] found an increase in challenging behaviors, such as agitation, in persons with dementia, while Cohen-Mansfield and Golander [67] noted a rise in hallucinations [67]. It can be assumed that behind challenging behaviors are unmet needs—expressed in the form of behaviors [68]. Unmet needs arise from a lack of reciprocity of perspective between people with dementia and their (social) environment [69,70]. In their findings, Cohen-Mansfield et al [34] showed three main unmet needs of persons with dementia exhibiting challenging behaviors: (1) boredom or sensory deprivation, (2) loneliness or a lack of social interaction, and (3) the need for meaningful activities.

To obtain an idea of times without care in persons with dementia, it seems necessary to examine the structures of these periods of time. According to the phenomenological or pseudophenomenological findings of Honer [71], the lifeworld understanding of time in persons with dementia is characterized by confusion between the subjective and intersubjective perceptions of temporal structures. Persons without dementia can switch their thoughts between worldtime-related present, past, and future. By contrast, persons with dementia confuse the worldtime-related past with the present, which affects their perception of here-and-now-reality. The comments by Honer [71] indicate that the temporal structures of the lifeworld of persons with dementia differ from those of persons without dementia. In addition, persons with dementia experience time subjectively differently [71].

Dealing with times without care and encounters requires an exploration of their origins, subjective experiences, and the subjective attribution of meaning. This applies particularly to those times that are intersubjectively described as long periods of boredom and as supposedly destructive. These are conditional social categories of time, whose effects are described from a rather objective perspective. Little is known about their genesis [72], temporal perception [73,74], and meaning for persons with dementia [75,76]—and for formal or informal caregivers—as well as their relation to the past and the future [77].

There is a need to investigate these times and to examine how persons with dementia perceive them. It is not known how to interpret times without care and encounters from the perspective of persons with dementia. This knowledge is particularly important, given the different definitions of the term *need for care* in the assessment of care and support conditions in Germany and Switzerland. In the Swiss and German care systems, different instruments are used to identify care needs and record care and support times. These instruments refer to national definitions of the need for care. While the German concept is based on the degree of independence and abilities of the care recipient, the Swiss concept is focused on their support needs [78].

These conceptual logics result in different care services. One can assume that these conceptual logics influence the existence and experience of times without care and encounters [79,80]. Nevertheless, it would be premature to assume that these periods are generally undesirable, empty times that are frequently associated with distressing experiences. The periods could also be considered relaxing off time, allowing for *healing* contemplation in a condensed time—in the sense of time for oneself [42].

Times without care and encounters in persons with dementia are the focus of our explorative and sequentially designed multimethods ethnographic study [81-84]. We examine these periods of time across dementia phases in different institutional as well as domiciliary care arrangements in Switzerland and Germany. We want to clarify how people with dementia perceive and experience these times. It is unclear whether these times represent meaningfully definable periods of time—in the sense of periods of action—for people with dementia. We would like to close this research gap by investigating the way in which people with dementia act (or do not act) during these times without care and encounters.

### Aim and Research Questions

The research objective of the study is to ethnographically reconstruct times without care and encounters from the perspective of persons with dementia themselves and from the point of view of formal or informal caregivers. For this purpose, we apply ethnographic methods [85-87].

We are thus exploring the different small social lifeworlds [88,89] of people with dementia.

In doing so, we investigate how people with dementia experience times without care and encounters in their respective lifeworlds. We are interested in investigating how people with dementia act in these times and what strategies they use to deal with these times.

These explorations deal with not only foreign world experiences but also familiar everyday phenomena. We therefore orientate ourselves to the methodological considerations of Honer [71] on lifeworld-analytical ethnography. Hitzler and Eisewicht [86] have developed these considerations further. In addition, focused ethnography according to Knoblauch [85] serves as guidance. Both ethnographic strands are methodologically pluralistic and presuppose reflexivity in the research process [90].

Lifeworld-analytical ethnography refers decidedly [86] to the mundane-phenomenological approaches of lifeworld analysis [91]. Therefore, we relate ourselves, among others, to the reflexive attempt of pseudophenomenology realized by Honer [71] for describing “demential world experiences”. Due to the cognitive limitations of persons with dementia, dementia-related world experience as a research subject is associated with considerable validity problems. Thus, we try to adopt a naïve point of view in the analysis. We attribute a pseudonatural status to the respective situations [92].

Furthermore, we conduct a supplementary survey to validate the interpretative typology developed from the ethnographic material. Specifically, we examine the provisions, regulations, and associated dimensions of the phenomenon of times without care and encounters in persons with dementia. With the survey, we also want to create a complementary information base. We hope to obtain additional information on the frequency, duration, and effects of times without care and encounters. This occurs particularly against the background of different national care systems, institutional and domiciliary arrangements, types of organizations, grade-mix specifications, and staffing principles for nursing homes [48,93,94].

With regard to the so far scarcely researched times without care and encounters, the fundamental question posed by Goffman [95] arises: “What is going on here?” Do persons with dementia address these time periods? If so, *how* do they talk about them? *How* do they experience and interpret these times? *How* do formal or informal caregivers experience and interpret these periods?

We are interested in exploring (across disciplines) how these periods of times without care and encounters are described (1) from the perspective of persons with dementia and (2) from the point of view of their formal or informal caregivers; we are also interested in (3) *whether* and *how* persons with dementia “appresent” (in the phenomenological sense) [96,97] these periods of time and how these times affect persons with dementia.

With regard to the individual perspectives, we explore the aspects outlined in Textbox 1.

**Aspects of individual perspectives explored in this study.**

**From the perspective of persons with dementia**

- *Whether* and *how* persons with dementia individually and subjectively perceive and describe times without care and encounters
- *Which* verbal and nonverbal techniques and expressions they use to communicate about these times
- *Which* interpretations, wishes, intentions, and interests they express
- *What* persons with dementia do and *in which way* during times without care and encounters, *how* they use these times, and *whether* or *how* they prioritize their activities

**From the point of view of the formal or informal caregivers of persons with dementia**

- *Whether* and, if so, *how* formal and informal caregivers perceive and describe times without care and encounters
- *Which* verbal and nonverbal techniques and expressions they use to communicate about these times
- *Which* interpretations, wishes, intentions, and interests they express
- *How* they respond to perceived times without care and encounters and to the actions of persons with dementia

**From the perspective of appresentation**

- *How* times without care and encounters affect the physical, cognitive, and emotional states as well as the social interactions of persons with dementia
- *How* this effect manifests itself

## Methods

### Overview

We are guided by a hermeneutic sociology of knowledge that is based on the fundamental assumptions of social constructivism. According to this perspective, social reality is constructed by the everyday actors themselves (ie, in this case, the person with dementia). Our aim is therefore to reconstruct the construction of periods of care and those without care of people with dementia and translate them into scientific “second-order constructions” [98].

The project is scheduled to last 36 months. The work packages proceed partly in parallel, although mostly at different times and always in interdisciplinary dialogue. We interrelate the results of this work in an ongoing, reciprocal process and then gradually integrate them [99,100].

To do justice to the research interest, we organize the project on an interdisciplinary and multiprofessional basis. Of particular interest is the transnational use of the respective competence profiles, which are closely linked to the research questions. Expertise in the fields of nursing science, gerontology, sociology, and ethnography is located at the Eastern Switzerland University of Applied Sciences (Switzerland), the Fulda University of Applied Sciences (Germany), and the University of Applied Sciences Furtwangen (Germany). Expertise in cognitive psychology is located at the University of Geneva (Switzerland).

The cooperation between different sites and professions seems extremely useful and synergetic. It allows us to combine methodological competences for sociological, psychological, and practical nursing issues with expert knowledge concerning interaction-enabled and communicative action. In addition, the transnational research consortium can address national and international realities and relevancies as well as differences and similarities with regard to the questions posed.

Our research teams at the Eastern Switzerland University of Applied Sciences, University of Applied Sciences Fulda, and University of Applied Sciences Furtwangen are focusing on the ethnographic exploration of times without care and encounters on site. Our research team at the University of Geneva is focusing on the cognitive psychological parameters and is involved in the interdisciplinary and transnational fieldwork as well as in the survey. The respective analyses take place across locations and countries.

**Figure 1:**
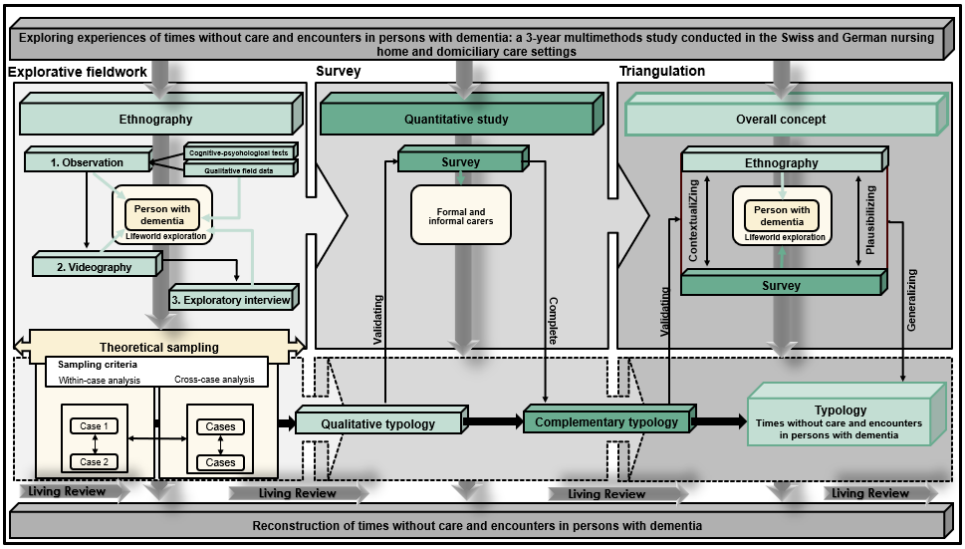
Project overview.

A combination of ethnographic observation and standardized survey procedures forms an adequate methodological approach to answer the research questions [101]. After the project installation, we explore the structures and dimensions of the lifeworld texture of time to appropriately reconstruct times without care and encounters in persons with dementia (Figure 1).

### Ethical Considerations

Ethics approval was granted by the responsible ethics committees (Switzerland: Ethics Committee of Eastern Switzerland [BASEC 2021-02150 and EKOS 21/179]; Germany: Ethics Committee of the German Society of Nursing Science: 21-009, BL 1787/1-1, and KO 5965/2-1).

Participation in the survey is voluntary for all participants (persons with dementia as well as informal and formal caregivers). We ensure that all participants receive comprehensive and target group–specific information. The project team is aware that persons with dementia are individuals considered potentially (highly) vulnerable [102]. Inclusion in the sample only occurs if it is possible to obtain a declaration of consent from authorized and legal representatives. However, if it is obvious that a person with dementia refuses to participate in the research project, we respect this—regardless of their legal representatives’ possible consent [79]. The design of the entire study and the proceedings ensure that participants face no exceptional risks. Participation takes place on the basis of ongoing consent [103,104]. All persons involved can withdraw from the project at any time. They can interrupt or cancel an observation. The designated research assistants have proven expertise in nursing and nursing science. They also have several years of research experience with persons affected by dementia. Therefore, they are able to pay sensitive attention to verbal and nonverbal signs of refusal or vegetative signs that may necessitate an immediate interruption or cancellation of the observation, interview, or video recording.

After the video recording, we review the footage with the persons with dementia and with their authorized representatives to confirm or revoke the previously granted release. However, our experience in previous projects shows that persons with dementia and their relatives use this option only occasionally—for various reasons.

In our view, it would be ethically unacceptable to pressure people to participate (and thus not respect their autonomy) or to withhold recordings from them.

It is crucial to ensure that the persons in question are aware of their option to codecide, to participate, and to access their data at any time—even later if they did not do so at an earlier stage. Being aware of the problems associated with videography involving persons considered (highly) vulnerable, we conduct prospective and retrospective ethical case discussions on the targeted video recordings. This occurs with the aim of ascertaining the presumed and situational will of the person with dementia and of complying with this will.

Against the background of (potential) vulnerability, we pay equal attention to providing comprehensive information to relatives about the voluntary nature of participation, the data collection methods, data protection, data analysis, data use, and data backup or archiving, as well as about the possibility of withdrawing consent and release.

### The Research Team

The persons responsible for the project and the research assistants have many years of experience in qualitative or interpretative and quantitative research. They are also experienced in the care and support of persons with dementia. To achieve the necessary intersubjectivity, we discuss and analyze all data in the interdisciplinary and cross-location team. For this purpose, we conduct ongoing interpretation meetings and workshops. We document all interpretation sessions and workshops, transcribe them into extracts, and compile them into a protocol.

Two nursing scientists advise and support the research team: Prof Dr Gabriele Meyer (Institute of Health and Nursing Science, Medical Faculty, Martin Luther University Halle-Wittenberg, Germany) and Prof Dr Hanna Mayer (Karl Landsteiner University of Health Sciences, Krems, Austria). Both have proven expertise in the field of dementia care research.

### Exploration

The ethnographic exploration and identification of times without care and encounters involves a close observation of these periods. We also observe periods that immediately precede and follow those without care and encounters. To capture the behaviors and strategies of persons with dementia in these periods of time in a methodologically sound way, it is necessary to (1) observe them both in a *participatory* and *nonparticipatory* manner, (2) video record them, and (3) talk to persons with dementia or their caregivers. This involves situational conversations and follow-up qualitative interviews.

To validate the developed typology and to obtain complementary information on times without care and encounters, we subsequently conduct a standardized survey using a self-developed instrument (refer to the Detailed Methodological Outline subsection). Formal and informal caregivers take part in this survey, and we analyze their written statements (refer to the Detailed Methodological Outline subsection). Afterward, we synthesize the knowledge generated, and the associated combination of qualitative and quantitative research results in an overall model of times without care and encounters in persons with dementia (refer to the Detailed Methodological Outline subsection).

### Research Field and Sample

We intend to collect data in the following settings:

- German and Swiss institutions in which persons *with* dementia live *separately* from those *without* dementia (segregation is explicitly envisaged as a concept [105])
- German and Swiss facilities in which persons *with* dementia and those *without* dementia live *together* (the concepts of integration and inclusion are explicitly envisaged)
- German and Swiss private households where persons with dementia reside [105]

In this study, we involve persons classified as dementia levels 3 to 7 according to the Global Deterioration Scale developed by Reisberg et al [106]. This means that we include persons with mild to severe loss of cognitive abilities.

As part of the observation procedures, we perform an initial screening by means of the Global Deterioration Scale [107]. We sample according to theoretical criteria and the principle of maximum and minimum contrast. Thus, we select people of different sexes and ages as well as with contrasting behaviors and care arrangements [108,109]. It is inherent to the strategy of theoretical sampling and maximum and minimum contrasting that the number of participants is determined during the research process. However, due to time constraints and the scope of the planned study, we plan 10 case observations per type of care. This corresponds to 30 case studies. The population of the planned survey (work package 3) includes formal and informal caregivers of persons with dementia in inpatient long-term care and domiciliary care settings in German-speaking Switzerland and Germany. We will analyze the data obtained through the survey using confirmatory factor analysis (CFA). While there are challenges in determining the statistical power and sample size for such an analysis [110], a widely accepted ratio is 10 cases per indicator variable [111]. We expect to generate approximately 50 indicator variables during the typology development, which would require a sample of 500 individuals. Accounting for a dropout rate of 10% [112], the recruitment of 50 additional individuals would result in a total required sample of 550 individuals. Given the difficulty of recruiting informal caregivers [113], we aim to recruit more formal than informal caregivers. We therefore plan to recruit an opportunity sample comprising approximately 400 formal (Germany: n=250, 62.5%; Switzerland: n=150, 37.5%) and 150 informal (Germany: n=100, 66.7%; Switzerland: n=50, 33.3%) caregivers.

### Detailed Methodological Outline

#### Work Package 1: “Preparation”

The first work package, “Preparation” (2 months), focuses on preparing, organizing, and installing project structures. It also allows a comparison of the working methods of the interdisciplinary, multiprofessional, and transnational research team members. This work package also includes targeted sampling for the domiciliary care setting.

#### Work Package 2: “Explorative Fieldwork”

##### Overview

In the second work package (24 months in total), we collect data in the research field. We process and analyze data in parallel. The eight steps that make up this work package are detailed in the following paragraphs: first, participatory observations (4 months) in the respective institutions and households serve to identify times without care and encounters and determine the institutional sampling. In terms of research practice, we familiarize ourselves with the field and with the persons living and working in these settings. In return, persons in the field can become familiar with our research assistants. To this end, research assistants take on care and support tasks, depending on their own expertise. In some cases, they take part in the everyday lives of persons with dementia. In doing so, we develop preliminary hypotheses about the interpretation and effects of times without care and encounters, as well as about strategies for dealing with them.

Second, by means of nonparticipatory observations (structured and unstructured; 4 months) [87] in the institutional setting and by further participatory observations in the domiciliary care setting, we continue to identify times without care. We explore strategies for dealing with these periods on site or in situ, with the aim of formulating statements that are appropriate to the subject matter; for example, we document the time, duration, and frequency of these occurrences, as well as physical and verbal patterns of appresentation [96,97] (in the phenomenological sense), alongside the behavior and actions of persons with dementia during these periods. In addition, we capture upstream and downstream social times and worldtime-related events. These should reveal both communicative regularities and remarkable irregularities in the interactions between formal and informal caregivers or among fellow residents. In doing so, we further test and specify the assumptions we have made so far.

Third, we plan to perform structured nonparticipatory observations using Dementia Care Mapping (DCM) [114,115]. In this way, we intend to uncover worldtime-related processes and social-worldly predefined limits of the time budget [71] and the forms or types of interactions. We also identify the impact of times without care and encounters. On the basis of DCM analysis data, we narrow down the subsequent unstructured nonparticipatory observations to the identified periods of time. By means of focused and multiday observations, we further explore the periods of interest in greater depth. We also talk to persons with dementia *during* and *after* an observation. In situational conversations [71], we find out *if,* *how,* and *to what degree* of awareness persons with dementia frame and describe the situation in the sense of the fundamental question posed by Goffman [95]. Of note, DCM only takes place in an institutional context, not in the domiciliary care setting.

Fourth, the time periods of interest and the associated communication or interaction processes are both highly complex and ephemeral. We assume that the researchers influence the experience of time merely by *being present*, whereas the exploring person as such represents an intervention [116]. On the basis of this assumption, it is necessary to consider how the researcher influences the field. It is essential to clarify whether questions can be derived from the observations: what if the exploring person had not been there? How do persons with dementia coconstruct the situation? How would they have dealt with the situation? How do persons with dementia comment on the situation? What is the result of the situation? Is it possible to anticipate how persons with dementia would have spent the time if the researcher had not been present? Inevitably, the person exploring the field becomes the subject (or object?) of the analysis. Therefore, we video record times without care and encounters (4 months). Using videography [117], we record the range of expressive variants appearing on micro- and nanolevels (language, facial expressions, gestures, body orientation, voice pitch, and melody, as well as their interplay in interactions). Videography takes place in a targeted manner—after careful consideration within a framework that seems acceptable to the participants involved. Despite its clear benefits, videography is an invasion of privacy—it interferes with the leisure time of persons with dementia and with the private spheres of formal or informal caregivers. For this reason, we use the camera sparingly, in a focused way, and only to the extent strictly necessary to obtain answers to our research questions. We conduct at least 2 video recordings for each person recruited (60 in total). The findings from our previous observations guide and determine the selection of interactions to be video recorded [118,119]. In the context of domiciliary care, we involve informal caregivers in a participatory way as coresearchers in the videography process—with the aim of reducing possible reactance to the camera [120].

Fifth, to reconstruct the respective subjective views and explicable background knowledge of the formal or informal caregivers, we conduct exploratory interviews [71] over a period of 4 months. We want to gain insights into the caregivers’ awareness of times without care and encounters. How do they understand these periods of time? Do they consciously use strategies to promote or reduce these periods? If so, which strategies do they use? In addition, we seek to find out the extent to which organizational framework conditions are beneficial or obstructive [121]. Which conditions are favorable or problematic according to the caregivers? Which options do they consider as desirable for solving difficulties in dealing with times without care and encounters? We orient ourselves toward the ideal of an interview that is minimally standardized but structured by means of a guideline. This is intended to ensure that the respondents are as open as possible in their responses, while at the same time maintaining the thematic focus of the interview [122,123]. We plan interviews, based on a semistructured interview guide [124], with at least 2 formal or informal caregivers per recruited person (60 interviews in total).

Sixth, in a further assessment step (parallel to steps 2 and 3), we use cognitive-psychological methods to determine the effect of times without care and encounters on the cognitive and emotional states of persons with dementia. Whenever possible, we use self-assessment tools for persons in the early stages of dementia. If self-assessment is impossible, data collection is based on proxies or an external assessment instrument. The repeated use of compact assessment tools (at least 4 assessments at 3-month intervals) allows us to document and examine changes. We focus on assessing the general cognitive state, emotional state, independence in activities of daily living, and perceived loneliness. We will use the Cognitive Telephone Screening Instrument [125] to assess the general cognitive state and the Geriatric Depression Scale [126] to evaluate emotional state in a global perspective; in addition, we will use the Short Depression-Happiness Scale [127] and the Self-Assessment Manikin scale [128] to capture momentary perspectives on emotional state. Furthermore, we will assess functional independence in everyday life (using the Index of Independence in Activities of Daily Living [129]) and perceived loneliness (using the De Jong Gierveld Short Scales for Emotional and Social Loneliness [130]). We also aim to evaluate mediators such as cognitive reserve (using the Cognitive Reserve Index questionnaire [131]) and desires or goals (by means of the Sense of Coherence questionnaire [132]). We will consider the social network of persons with dementia and their contact with this network [133]. Moreover, we will measure stress using the abbreviated Perceived Stress Scale [134].

Seventh, in the second phase of data collection (4 months), we conduct observations and video recordings. We also apply the knowledge gained from previous observations and interviews to currently observed situations. In this way, we clarify questions that remain unanswered. This relates to, for example, our more precise understanding of persons with dementia and their formal or informal caregivers. During this phase, we also perform observations at different times of the day. Whether it will be necessary at this point to focus specifically on certain times without care and encounters will depend on the findings obtained up to this point. We process and analyze the data collected in this stage and relate it to the analyses carried out so far.

Eighth, after the phase of direct fieldwork (4 months), we interrelate, compare, and contrast the findings and assumptions from the analyses of observations and interviews as well as video data and neuropsychological assessment data collected at different points of time.

##### Qualitative Data Analysis

We ensure the trustworthiness and credibility of our research by using a comprehensive process of documentation, an interpretation practice based on intersubjectivity, a uniform rule-based approach, a (trusting) proximity to the object of the research, a plurality of methods in data generation and analysis (ie, triangulation), and the continual reflection of the involved researchers [135].

To combine sampling, data collection, interpretation, and theory formation, we use grounded theory [109] as a corresponding [89] research approach in the sense of a hermeneutic grounded theory [136]. We document all observations in protocols and transcribe the audio data. After each step of data collection, we perform a successive overall review of the data which are initially open-coded, then axially coded, and finally selectively coded [109,137]. In addition, we deliberately select certain passages and subject them to additional hermeneutic sequence analysis [138-143]. In doing so, we relate the different observation situations and the interview statements to each other and compare them. Supplementary video data provide the necessary access to the complexity of the events of interest. They allow an in-depth, repeated evaluation by means of video interaction analysis [117,144]. This is of central importance for clarifying the questions raised in this context.

Our experience in analyzing video-recorded care interaction situations revealed that this method indeed allows us to capture important subtleties of communication and interaction that would otherwise have remained hidden. During the joint cross-site and cross-national interpretations [145], it is necessary to implicitly and explicitly triangulate different types of qualitative data with regard to their meaningful referential context [146]. We clarify *whether* and *how* they correspond, complement, or contradict each other. Furthermore, we retrace the referential contexts of events and actions thematized in the interviews and conversations. We also backtrack to events and actions that emerged in the participatory or nonparticipatory observations and in the video documentation.

#### Work Package 3: “Survey of Times Without Care and Encounters”

##### Overview

In this work package (14 months in total), we collect, edit, and analyze quantitative data. The design we chose is a standardized mixed modes survey (eg, web-based survey tool and paper-pencil version) directed at formal or informal caregivers. The questionnaire is based on the results of the ethnographic observations and the interviews. To test the questionnaire, we use a qualitative pretest procedure with expert validation [147]. The analysis involves multivariate statistical methods. The methodological approach, which involves five steps, is detailed in the following paragraphs: first, we develop the item batteries and case vignettes for the survey (over a 6-month period)—based on the elaborated typology and the associated phenomenal dimensions. This takes place in parallel and in coordination with work package 2. We test the instrument with 15 participants in a qualitative pretest procedure (interviews; 3 months) and adapt it accordingly. Participants must be aged at least 18 years to be included in the interview. We aim to ensure diversity among the participants, particularly regarding their educational backgrounds, professional experiences, and other relevant demographic factors (eg, sex and age). This approach intends to capture a wide range of perspectives and ensure a more comprehensive understanding of the topic. The project team is responsible for recruiting participants, with recruitment efforts coordinated to ensure diversity. We ask the participants to think aloud while filling in the survey (ie, the thinking-aloud method). The interviewer (ie, project member) will document these thoughts in protocols. Upon survey completion, participants are asked standardized questions (eg, whether there are terms they do not understand and whether the length of the survey is appropriate). The interviewer documents the answers in written form, and a project member evaluates the protocols. We discuss potential adjustments to the survey within the team before implementation.

Second, sample recruitment (3 months) occurs as part of an ad hoc procedure. To this end, we develop and implement a gatekeeper [148] system for recruiting in the institutional sector (nursing homes) and in the outpatient sectors (domiciliary care and self-help organizations, eg, Alzheimer Switzerland and the German Alzheimer Society). The target sample consists of formal and informal caregivers of people with dementia in Switzerland and Germany. To meet the inclusion criteria, caregivers must be aged at least 18 years. Formal caregivers should work in the institutional long-term care setting. These are the only criteria for inclusion.

Third, the survey (5 months) is designed as a mixed modes survey (web-based, written-postal, and telephone). Qualtrics (Qualtrics International Inc) will be used as the web-based survey platform [149].

##### Quantitative Data Analysis

The analysis (2 months) is based on descriptive and inferential statistical methods. First, we will analyze the survey data descriptively, thereby providing information on the sample characteristics (eg, the mean age of the study sample). Using CFA, we will test whether the model (hypothesized typology) fits our collected data. CFA belongs to the broader family of structural equation modeling and is typically used to validate the relationships between observed variables (indicators, ie, survey items) and underlying latent variables (factors, ie, typology) that are not directly measured [150]. Our null hypothesis is that the model (typology) will fit the data well, with the specified factor structure (or typology) accurately reflecting the real-world phenomena captured by our data. The alternative hypothesis posits that the proposed model does not fit the data, suggesting that the typology does not adequately represent the real-world phenomena and fails to capture the underlying constructs or relationships, as reflected in our data. CFA tests these hypotheses using model fit indices, such as the root mean square error of approximation or comparative fit index [150]. These indices help evaluate whether the null hypothesis (good fit) is reasonable or whether the alternative hypothesis (poor fit) is supported. A well-fitting model will have values within the acceptable ranges for these indices.

#### Work Package 4: “Overall Concept and Dissemination”

A triangulation focusing on convergence and complementarity [151] takes place in the fourth work package (6 months in total). For this purpose, we integrate the results of the ethnographic research and the survey. We analyze the results in cross-site, web-based group interpretations to determine whether and how they correspond, complement, or contradict each other. This occurs with the aim of contextualizing, plausibilizing, and, if necessary, generalizing the elaborated typology. The triangulation strategy paves the way to the concentrated final phase aimed at developing the overall theoretical concept for times without care and encounters in persons with dementia.

#### Work Package 5: “Living Literature Review”

A living literature review accompanies the project. The detailed methods are described elsewhere [152]. The specific aim of this review is to provide a continually updated overview of times without care and encounters from the perspective of persons with dementia and formal or informal caregivers. This can have a guiding function for data synthesis and interpretation. During the entire project, we conduct comprehensive systematic literature searches in MEDLINE, PubMed, CINAHL, PsycInfo, Ovid, and Web of Science Core Collection (citation-based searches and web searches). We include observational studies performed in the institutional or domiciliary care setting and published in English, French, or German, without restrictions on the year of publication. Studies have to address times without care and encounters from the perspective of persons with dementia and formal or informal caregivers. One reviewer screens titles, abstracts, and full texts and extracts data. We will present the key characteristics and results of the included studies in tabular form. Searches take place during the entire study (every 6 months for 2 years; 2023-2025) [152].

## Results

The study started in March 2022 and ends in May 2025. It is underway as part of a lead agency process and has been funded by the Swiss National Science Foundation (200919) and the German Research Foundation (458561353).

The exploratory investigations associated with work package 2, the within-case analyses, and the location-related and cross-country cross-case analyses concluded in September 2024. The cross-national survey results will be available in December 2024. Work packages 4 (“Overall concept and dissemination”) and 5 (“Living literature review”) are scheduled for completion in April 2025.

## Discussion

### Summary

Our approach enables us to obtain information about the nature, frequency, and prevalence of the phenomenon of times without care and encounters. We will be able to make statements about how people with dementia and their caregivers experience and interpret these times for themselves. We will demonstrate how people with dementia act during these times and what purpose they pursue. The developed typology will provide the foundation for assessing these periods using a survey instrument, which can be validated in a follow-up project, with two intentions: (1) to assess times without care and encounters in a representative and reliable way with this instrument and (2) to develop a diagnostic instrument for nursing practice.

Furthermore, with this project, we would like to make a fundamental methodological contribution to advancing research *on* and *with* persons with dementia.

In the context of dementia care research, procedures for field research, intervention planning and implementation, and data collection require an extended framework of both care ethics and research ethics. However, at the same time, there are almost insurmountable problems of validity and generalization. The proposed research strategy envisages not only methodological and methodical triangulation but also data triangulation and researcher triangulation. In this way, we would like to contribute to the existing cross-disciplinary discourse on these problems. However, our research is limited by the fact that we are studying 2 relatively similar care cultures and cannot resolve validity issues. Nevertheless, this research represents an ongoing effort to understand and reconstruct the lifeworld of people with dementia.

### Conclusions

With our research, we intend to pave the way for a qualitative assessment of lived times without care and encounters in persons with dementia and for scrutinizing organizational and professional practices. In doing so, we also ask about the need for change. However, expecting a priori a need for change with regard to all times without care and encounters seems to be inappropriate and paternalistic—despite initial scientific findings pointing in this direction. Therefore, it is our intention to present examples that have been described and proven as good practice.

In this research project, we shed light on the concepts of autonomy, well-being, independence, self-determination, and meaningful activities of persons with dementia in relation to the need for security and continuous attachment to formal and informal caregivers in times without care and encounters. The tensions involved in everyday dementia care will continue to intensify as social and human care resources become increasingly scarce. Against the background of the current political and social understanding of care, it is unlikely that the situation regarding times without care and encounters in persons with dementia, although considered worthy of change, will be addressed in a humane way. In a survey of nursing and care staff in Swiss care institutions, caregivers reported times when they had to keep residents waiting or were unable to offer them emotional support or engaging care [153]. Therefore, it seems essential to consider alternative interventions and possibly technical alternatives.

## Appendix

Multimedia Appendix 1 Peer review report by the Humanities and Social Sciences (Division I) - Swiss National Science Foundation (SNSF) (Switzerland).

## Abbreviations

CFA: confirmatory factor analysis
DCM: Dementia Care Mapping

## Data Availability

Data sharing is not applicable to this paper as no datasets were generated or analyzed during this study. The results data will be deposited in publicly available repositories as requested by the funding agency (Swiss National Science Foundation).
