# Supplementary material for: Exploring the Experiences of Times Without Care and Encounters in Persons With Dementia in the Swiss and German Nursing Home and Domiciliary Care Settings: Protocol for an Ethnographic Multimethods Study
Source: JMIR Res Protoc. 2024 Nov 18;13:e58190. doi: 10.2196/58190 (PMC11612575; doi:10.2196/58190)
Supplement: Multimedia Appendix 1 [file resprot_v13i1e58190_app1.pdf]

Review: 1

Application data

Applicant(s)

Beer, Thomas

Bleses, Helma M. / Kliegel, Matthias / König, Peter / Misoch, Sabina

Betreuungs- und begegnungsfreie Zeiträume: Methodenplurale Erkundungen zum Erleben von Personen mit Demenz

Project funding in humanities and social sciences (division I)

Detailed evaluation

Applicants' scientific track record and expertise

The applicants represent different scientific fields and traditions. Among them, Prof. Misoch and Prof. Kliegel are emminent representatives in their field concerning their CV and scientific output. Other applicants presents a more restricted scientific production. The individual comepetence and scientifc output ranges from excellent to moderate. Some applicants have own extensive expierence in nursing, dementia research and qualitative research. This might be an advantage in this specific research field. There seems to be some "underuse" of empirical-statistical research expertise, which is present in the consortium.

Scientific relevance, originality and topicality

The research question of the proposal is interesting and has gained actuality with the question of isolation of vulnerable groups in the context of infectious diseases. The approach to establish a typology of (experienced) time alone of people with dementia is explorative.

Broader impact (forms part of the assessment of scientific relevance, originality and topicality)

The topic of the research has gained actual interests, because questions of ethics and resource distributions are becoming more important in the ageing European societies. The project may produce different "narratives" concerning the time, people with dementia spend alone. The impact on a validated measurment of experience of time by people with moderate to severe dementia may be low. The research may increase the societal attention to social inclusion of people with dementia.

Suitability of methods and feasibility

The methodological approach incorporates some novelities, e. g. video assessments. Long phases of video documentation or other forms of automatic monitoring may indeed add new information of the development of time allocation in groups and time spent alone. The authors mentioned the issues of personality rights in this context. This might be a major concern of ethics comittees or employees in nursing homes. However, the restriction of the use of video to approx. 60 short video sequences seems to be very restricted. The main corpus of data are observational data from appr. 30 persons with moderate to severe dementia in three different settings. This approach stands in a long tradition of observation with and without participation. There is a high risk that heterogenity in people with dementia, dementia state and settings will interfere with the construction of a typology of the self experience of lonesomeness/loneliness of people with dementia. The applicants themself have raised the question of validity and generalisability. The suggested triangulation of different research perspectives may help only a little bit.

Comment

The applicant tackles an interesting topic in dementia care. The question, how people with dementia experience times without activities is important for ethical descisions concerning resource allocation in dementia care. The quantitative research seems interessting and the use of video observation as a new tool to quantify behaviour in the absence of other persons may bring new inside into the use of time in dementia. The qualitative approach does not realy compensate for the problem, that the first person perspective in severe dementia is not easily "experienced" by observation with/without participation.

Note on the evaluation procedure

The evaluation bodies of the SNSF strive to reach a balanced overall assessment of each proposal. External reviews play an important role in this. Reviewers generally review only one proposal. The evaluation bodies of the SNSF, however, must compare and rate the quality of all proposals submitted by a given deadline. The opinions expressed in external reviews are generally positive, or they may occasionally include critical remarks that are largely irrelevant to the assessment conducted by the evaluation body. Therefore, the final decision taken by the SNSF evaluation bodies need not necessarily reflect the content of external reviews.

Review: 2

Application data

Applicant(s)

Beer, Thomas

Bleses, Helma M. / Kliegel, Matthias / König, Peter / Misoch, Sabina

Betreuungs- und begegnungsfreie Zeiträume: Methodenplurale Erkundungen zum Erleben von Personen mit Demenz

Project funding in humanities and social sciences (division I)

Detailed evaluation

Applicants' scientific track record and expertise

Kliegel
Publikationen / Forschungsoutput:
zahlreiche (internationale) Veröffentlichung, (auch) in Zeitschriften mit peer-review-Verfahren
excellent

Wissenschaftskommunikation und -vernetzung:
Organisation zahlreicher (internationaler) Konferenzen etc., Preisträger, Herausgeber eines wissenschaftlichen Journals
excellent

Fachkompetenz in Bezug auf das Forschungsvorhaben:
Die Fachkompetenz von Herrn Prof. Kliegel in Bezug auf das Forschungsvorhaben wird als exellent beurteilt, was er nicht zuletzt durch seine Professur der Gerontopsychologie und der Leitung des CIGEV belegt. Besonders hervorzuhaben ist außerdem die überaus erfolgreiche Durchführung von drittmittelgeförderten Forschungsprojekten.

Bleses
Publikationen / Forschungsoutput:
keine aktuellen Veröffentlichungen in peer-reviewten Zeitschriften, aber in Sammelbänden
gut

Wissenschaftskommunikation und -vernetzung:
keine Organisation von Tagungen, kaum eigene Beiträge bei Tagungen
mäßig

Fachkompetenz in Bezug auf das Forschungsvorhaben:
Einwerbung und Durchführung von drittmittelgeförderten Forschungsprojekten, Inhaberin der Professur für Pflegewissenschaft und klinische Pflege
sehr gut

Beer
Publikationen / Forschungsoutput:
zahlreiche (internationale) Veröffentlichung, (auch) in Zeitschriften mit peer-review-Verfahren
excellent

Wissenschaftskommunikation und -vernetzung:
eigene Beiträge bei Tagungen etc., regelmäßige Organisation von Konferenzen
excellent

Fachkompetenz in Bezug auf das Forschungsvorhaben:
Inhaber der Professur für Pflegewissenschaft, Zusammenarbeit mit Prof. Bleses in Drittmittelprojekten; keine eigenständige Einwerbung eigener Projekte
gut

Misoch
Publikationen / Forschungsoutput:
einige (internationale) Veröffentlichung, auch mit peer-review-Verfahren
sehr gut

Wissenschaftskommunikation und -vernetzung:
eigene Beiträge auf Tagungen, Leiterin von AGE-NT
excellent

Fachkompetenz in Bezug auf das Forschungsvorhaben:
Professorin und Leiterin des Instituts für Altersforschung, eigenständige Einwerbung und Durchführung von Drittmittelprojekten
excellent

König
Publikationen / Forschungsoutput:
einige (internationale) Veröffentlichung, auch mit peer-review-Verfahren
sehr gut

Wissenschaftskommunikation und -vernetzung:

Fachkompetenz in Bezug auf das Forschungsvorhaben:
Inhaber der Professur für Pflege und Rehabilitationsmanagement, eigenständige Einwerbung und Durchführung von Drittmittelprojekten
excellent

Beurteilung des Forschungsteams
Das Forschungsteam wird als *sehr gut* dazu geeignet betrachtet, das mehrperspektivische "methodenplurale" Forschungsvorhaben umzusetzen.

Scientific relevance, originality and topicality

Wissenschaftliche Bedeutsamkeit
Das Forschungsvorhaben wird als wichtige Erweiterung des Kenntnisstandes im Fachgebiet betrachtet. Es hat das Potenzial, das Verstehen rund um Demenz zu erweitern. Davon gibt auch der akribisch ausgearbeitete Stand der Forschung Zeugnis.

Originalität und Aktualität
Die, wie die Antragstellenden es nennen, "methodenplurale" Herangehensweise im Forschungsvorhaben ist tatsächlich originell und verspricht breite Erkenntnisse zu liefern. Auch die zugrundeliegenden Forschungsfragen sind, meines Wissens nach und so wie es die Antragstellenden ausgeführt haben, bislang nicht - und vor allem nicht in dieser methodischen Art und Weise - untersucht worden, was gleichzeitig Auskunft über die Aktualität des Vorhabens gibt. Diese wird, was sehr anschaulich und nachvollziehbar ist, außerdem durch den Bezug auf die Auswirkungen der Corona-Pandemie verdeutlicht. Gleichzeitig hätte in den Ausführungen zum Stand der Forschung tw. noch mehr Bezug auf aktuelle(re) Veröffentlichungen genommen werden können.

Broader impact (forms part of the assessment of scientific relevance, originality and topicality)

Demenz ist eine Lebensphase, die wir potenziell alle eines Tages durchleben können. Von daher ist die außerwissenschaftliche Bedeutsamkeit der Ergebnisse, die das Forschungsvorhaben zu liefern verspricht, sehr hoch. Hervorgehoben werden kann hier als ein Beispiel die hohe Bedeutung der Ergebnisse für die Handlungspraxis, die jedoch möglicherweise erst einmal nicht unbedingt Einsicht in die zugrunde liegende Problematik zeigt. Dies aufzufangen ist eine der weiterführenden Herausforderungen der geplanten Studie.

Suitability of methods and feasibility

Das geplante methodenplurale Vorhaben nimmt sich viel vor und es ist sicherlich eine große Herausforderung, die wirklich zahlreichen unterschiedlichen Herangehensweisen 'unter einen Hut zu bringen'. Die Antragstellenden haben sich dazu verschiedene Maßnahmen auferlegt, anhand derer ein Austausch und eine interdisziplinäre Reflexion stattfinden sollen (z.B. die genannten Workshops), was positiv bewertet wird.
Es stellt sich die Frage, weshalb der Survey (standardisierte Befragung mittels eines selbstentwickelten Instruments) erst anschließend an die Typologisierung durchgeführt wird. Insbesondere die Idee der 'Überprüfung und komplentären Informationsgewinnung' erscheint als nachträgliche 'Weihung' der Ergebnisse und sollte hinterfragt werden. Warum nicht mit dem Survey beginnen und so das Feld eröffnen?
Dennoch sind die Methoden geeignet, die Fragestellungen zu beantworten (lediglich die Reihung sollte meines Erachtens überdacht werden).
Da ausreichend Gelder für wissenschaftliche Mitarbeit und Hilfskräfte beantragt werden, wird davon ausgegangen, dass das Vorhaben in der vorgesehenen Zeit und mit den zur Verfügung stehenden personellen Ressourcen bewältigt werden kann. Die klar genannten 'Meilensteine' strukturieren das Vorhaben und sind sinnvoll gesetzt. Positiv hervorzuheben sind außerdem, erneut, die sogenannten Workshops zur Methoden- und Ergebnisdiskussion.

Comment

Stärken sind: Erfahrungen in der Zusammenarbeit und in interdisziplinären Projekten; äußerst wichtige und bislang kaum berücksichtigte Fragestellungen; interessante und vielversprechende multiperspektivische Herangehensweise, die allerdings gleichzeitig eine Schwäche sein kann, wenngleich die Antragstellenden Mechanismen gefunden haben, wie sie die Kooperation steuern und verwirklichen wollen.

Note on the evaluation procedure

The evaluation bodies of the SNSF strive to reach a balanced overall assessment of each proposal. External reviews play an important role in this. Reviewers generally review only one proposal. The evaluation bodies of the SNSF, however, must compare and rate the quality of all proposals submitted by a given deadline. The opinions expressed in external reviews are generally positive, or they may occasionally include critical remarks that are largely irrelevant to the assessment conducted by the evaluation body. Therefore, the final

decision taken by the SNSF evaluation bodies need not necessarily reflect the content of external reviews.

Review: 3

Application data

Applicant(s)

Beer, Thomas

Bleses, Helma M. / Kliegel, Matthias / König, Peter / Misoch, Sabina

Betreuungs- und begegnungsfreie Zeiträume: Methodenplurale Erkundungen zum Erleben von Personen mit Demenz

Project funding in humanities and social sciences (division I)

Detailed evaluation

Applicants' scientific track record and expertise

Alle am Antrag Beteiligten sind wissenschaftlich sehr gut bis exzellent ausgewiesen. Die Zusammensetzung des Teams ist einleuchtend angesichts der unterschiedlichen fachlichen Spezifikationen, die im Team vertreten sind.

Scientific relevance, originality and topicality

Die Fragestellung ist wichtig und innovativ. Es gibt kaum Studien zur vorgeschlagenen Thematik. Dass das Thema Demenz von großer gesellschaftlicher Gegenwartsbedeutung ist, ist evident. Die Folgen der Coronaepidemie für Menschen mit Demenz sind gerade deutlich geworden, sodass die Frage nach den Zeiten, in denen MmD ohne Zuwendung sind, von erhöhter Bedeutung ist.

Das beantragte Forschungsprojekt ist bedeutsam, denn das Thema Demenz wird zur zentralen Herausforderung in mitteleuropäischen Gesellschaftern.
Das beantragte Forschungsprojekt wist originell, denn es gibt kaum Studien zur Lage von Menschen mit Demenz ihn betreuungsfreien Zeiten.
Das beantragte Forschungsprojekt ist aktuell, weil die Pflegesituation zunehmend auf einen Notstand hinausläuft - ganz abgesehen davon, dass die Coronapandemie neue schwere Beschädigungen bei Menschen mit Demenz heraufbeschworen hat.

Broader impact (forms part of the assessment of scientific relevance, originality and topicality)

Das Thema des Projektes könnte von großer ausserwissenschaftlicher Bedeutung sein. Ein so umfangreiches und aufwändiges Projekt allerdings sollte m.E. deutlicher als geschehen die Frage einbeziehen, was denn nun, wenn die Sichtbarmachung der Lebenswelt von Personen mit Demenz gelingt, in diesen besser ausgeleuchteten Räumen geschehen soll.

Angesichts der Kompetenz und Prominenz der beteiligten Wissenschaftlerinnen scheint mir ein blinder Fleck in der Antragstellung bedauerlich, der allerdings nicht zufällig ist. Die perfekte Präsentation der wissenschaftlichen Instrumente lässt eine kritische Reflexion des Vorhabens nicht deutlich erkennen. Der Blick auf die betreuungsfreien Zeiten kann ja auch im Foucaultschen Sinne als ein Beitrag zur Optimierung der Transparenzgesellschaft begriffen werden. Der Ausleuchtung der betreuungsfreien Zeiten dürften Konzepte folgen wie diese als leer diagnostizierten Räume gefüllt werden sollen und können. Angesichts der Feststellung, dass schon jetzt der Personalmangel die Zuwendungszeit deutlich begrenzt, wird sich am Ende des Projektes erkennen lassen, dass die unbetreute Zeit wissenschaftlich besser ausgeleuchtet ist, dass aber in der Praxis daraus keine Konsequenzen gezogen werden können. Warum also wollen wir das wissen, wenn doch nichts daraus folgt? Sind wir sicher, dass die Ausleuchtung der umbetreuten Zeiten den Betroffenen dienen wird? Wissenschaft hat sich der Ausleuchtung des Halbschattens verschrieben. Das geht latent auch immer mit Kontrolle und Überwachung einher - das hat Foucault immer wieder analysiert. Kann man sicher sein, dass die Aufhellung der dunklen Bereiche der Demenz denen dient, die sich in diesem Halbschatten des Bewusstseins aufhalten. Was untersucht, vermessen, quantifiziert, erfasst und interpretiert wird, ist immer auch in der Gefahr dem kolonisierenden Zugriff ausgeliefert zu werden. Wenn die Menschen mit Demenz nur noch als belieferungsbedürftige Mängelwesen wahrgenommen werden können, dann verschwindet ihr Eigenes endgültig. Ich hätte mir gewünscht, dass dieses für den Antrag zentrale Element der Herstellung von Transparenz mit ein paar deutlich ambivalent-kritischen Gedanken bedacht worden wäre.

Suitability of methods and feasibility

Die gewählten Methoden erscheinen mir angemessen, reflektiert und machbar. Sie passen zu den empirischen Settings und zu den Kompetenzen der Beteiligten Personen.

Comment

Die wissenschaftlichen Leistungen und Fachkomepetenzen der AntragstellerInnen sind sehr gut bis exzellent, sie gehören im Wesentlichen in eine eher praktisch, bisweilen affirmative Forschungstradition - dies indessen mit hervorragenden Ergebnissen.
Das beantragte Projekt ist bedeutsam, originell und hochgradig aktuell. Die Methoden sind einleuchtend und gut begründet Eine deutlicher kritische Reflexion der Frage, wie das hier formulierte wissenschaftliche Interesse den Wünschen der Menschen mit Demenz Menschen dient, wäre wünschenswert gewesen. Was bringt die Transparenz und was beschädigt sie unter Umständen?
Ich würde meine Beurteilung im Bereich zwischen 'exzellent' und 'sehr gut' ansiedeln.

Note on the evaluation procedure

The evaluation bodies of the SNSF strive to reach a balanced overall assessment of each proposal. External reviews play an important role in this. Reviewers generally review only one proposal. The evaluation bodies of the SNSF, however, must compare and rate the quality of all proposals submitted by a given deadline. The opinions expressed in external reviews are generally positive, or they may occasionally include critical remarks that are largely irrelevant to the assessment conducted by the evaluation body. Therefore, the final decision taken by the SNSF evaluation bodies need not necessarily reflect the content of external reviews.

Review: 4

Application data

Applicant(s)

Beer, Thomas

Bleses, Helma M. / Kliegel, Matthias / König, Peter / Misoch, Sabina

Betreuungs- und begegnungsfreie Zeiträume: Methodenplurale Erkundungen zum Erleben von Personen mit Demenz

Project funding in humanities and social sciences (division I)

Detailed evaluation

Applicants' scientific track record and expertise

Wissenschaftliche Qualifikation der Gesuchstellenden, Leistungsausweis und individuelle Fachkompetenz

Prof. Dr. Thomas Beer ist promovierter Gesundheits- und Pflegewissenschaftler und kann auf praktische Berufserfahrung als exam. Krankenpfleger zurückgreifen, was für die anvisierten Übertrag in pflegerische Handlungspraxis von Vorteil sein wird. Er hat als Leitung einer Einrichtung für Menschen mit Demenz auch in diesem Teilbereich pflegerischer Arbeit Erfahrung sammeln können; er beschäftigt sich seit 7 Jahren in Forschung und Lehre (u.a. in der Weiterbildung) mit der Vermittlung von Kenntnissen über Demenz. Projekterfahrung hat er zum Themenfeld des Einsatzes robotischer Assistenzsysteme in der Altenpflege mit Schwerpunkt auf Menschen mit Demenz gewonnen, was vor allem für die anvisierten nächsten Schritte der Projektidee interessant sein dürfte.

Als ausgezeichnet sind seine Kenntnisse in der Lebensweltlichen Ethnographie zu bezeichnen, die er mit den Begründern Anne Honer und Ronald Hitzler gemeinsam durchgeführt hat. Die Anwendung und Weiterentwicklung im Hinblick auf Kommunikation mit Menschen mit Demenz zeichnet ihn als besonders kompetent hinsichtlich der Aufgabe aus, die Sicht der Teilnehmenden zu rekonstruieren. Seine Vorarbeiten zeigen eine langjährige wissenschaftliche Beschäftigung mit dem Thema.

In den letzten fünf Jahren hat er sechs Publikationen in Erst- und 16 in Mitautorenschaft erstellt, die alle mit der Thematik Menschen mit Demenz und ihr Erleben bzw. pflegerische Angebote für diese Personengruppe befasst sind, auch hier mit dem Schwerpunkt auf robotischen Assistenzsystemen.

Prof. Dr. Helma Bleses hat vor ihrer akademischen Karriere langjährig in Pflege bzw. Pflegemanagement gearbeitet. Ihre beruflichen Schwerpunkte liegen nach wie vor im Bereich des Pflegemanagements und der Qualitätsentwicklung. Sie ist langjährig Studiengangsleitung mehrerer pflegewissenschaftlicher Studiengänge und konzipiert aktuell ein Masterprogramm, engagiert sich somit eindrucksvoll für die fachliche Ausbildung des Nachwuchses sowohl für die Pflegepraxis wie auch für die Pflegeforschung. Diese Kombination ihres fachlichen Engagements lässt vor allem erwarten, dass der alltagspraktische Nutzen und mögliche Umsetzung in die Praxis nicht aus dem Auge verloren werden, somit die außerwissenschaftliche Bedeutsamkeit dieses Projektes entfaltet wird.

Projekterfahrung hat sie vor allem auf dem Feld robotischer Assistenzsysteme und Kommunikation mit Menschen mit Demenz in zahlreichen, von ihr geleiteten Projekten gesammelt; auch Prof. Dr. Bleses ist ausgewiesene Expertin in der Lebensweltlichen Ethnographie.

Seit 2015 hat sie 4 Publikationen in Erst- und 10 in Mitautorenschaft veröffentlicht, mit dem

Schwerpunkt auf robotischen Assistenzsystemen in der Pflege von Menschen mit Demenz.

Prof. Dr. Matthias Kriegel ist habilitierter Universitätsprofessor für Gerontopsychologie, somit der Experte für das mit quantitativen Methoden zu erhebende und auszuwertende Survey zur Einschätzung von Pflegenden. Er hat seit 2007 Universitätsprofessuren im Bereich der Psychologie inne und ist Direktor des Interfakultären Forschungszentrums für Gerontologie und Vulnerabilitätsforschung, Präsident der Schweizer Gesellschaft für Psychologie und ein herausragender Experte seines Fachgebiets.

Er verfügt über eine breit angelegte Forschungspraxis in internationalen Projekten, mit unterschiedlichen gerontopsychologischen Schwerpunkten, und ist in der internationalen wie interdisziplinären Zusammenarbeit erfahren; im Zusammenhang des hier geplanten Projektes ist vor allem seine Expertise in Fragen von Kognition und Alter(n) sowie hinsichtlich des Einflusses von Lebensstilfaktoren hervorzuheben. Er hat international anerkannte Modelle und darauf aufbauende Testverfahren entwickelt, die dem Projekt zugutekommen werden.

Er kann auf eine eindrucksvolle Liste erfolgreich abgeschlossener Projekte verweisen (11 seit 2014). Auch seine sonstigen Tätigkeiten und Mitgliedschaften weisen ihn als international anerkannten Experten auf seinem Gebiet aus.

Entsprechend umfangreich ist die Liste seiner Publikationen, die eine umfängliche Dissemination der Projektergebnisse erwarten lässt.

Prof. Dr. Peter König ist promovierter Pflegewissenschaftler, seit 2012 an der Fachhochschule Furtwangen, und war nach seiner Ausbildung zum exam. Krankenpfleger bis zu diesem Zeitpunkt in der Pflegepraxis, zuletzt als Pflegedienstleiter, tätig. Er ist unter anderem Studiendekan im Masterstudiengang Angewandte Gesundheitsförderung und stellvertretender Vorsitzender der lokalen Ethikkommission, was auf entsprechende Expertise schließen und die Beachtung strenger forschungsethischer Richtlinien in diesem Projekt mit vulnerablen Personen, die einigen Belastungen ausgesetzt werden, vertrauen lässt.

Auf dieses Projekt bezogene Forschungsprojekte sind vor allem im Bereich der Erinnerungsarbeit mit Menschen mit Demenz zu finden, sonstige Tätigkeiten und Mitgliedschaften zeigen Prof. Dr. Königs Engagement im Bereich praxisnaher Forschung, wie seine Beschäftigung mit Fragen der Evidenzbasierung und der Leitlinienarbeit. Ein weiterer Forschungsschwerpunkt ist der Einsatz von technischen Systemen in der Pflege; er ist Vorstand des Forschungsinstituts Mensch, Technik und Teilhabe der Hochschule Furtwangen. Bezogen auf Menschen mit Demenz forscht er vor allem im Bereich der Biographiearbeit.

Seine Publikationsliste weist 3 Artikel in Erst- und 12 in Mitautorenschaft auf, mit einem Schwerpunkt auf der Erforschung technikgestützter Systeme.

Prof. Dr. Sabina Misoch ist promovierte Soziologin und Professorin an der Fachhochschule ST. Gallen, wo sie auch das Institut für Altersforschung leitet. Zuvor forschte sie am Institut für Qualitative Sozialforschung in Luzern, die letzten beiden Jahre als Leiterin. Das, wie auch ihre entsprechenden Veröffentlichungen, sprechen für eine exzellente Expertise und hohe Qualität des Methodeneinsatzes in diesem in der Hinsicht sehr anspruchsvollen Projekt.

Sie kann seit 2017 drei abgeschlossene und drei laufende Projekte vorweisen, die sich mit Techniknutzung und Alter(n) befassen. Auch ihre Mitgliedschaften und sonstigen Tätigkeiten zeugen von ihrem Engagement für diese Thematik, ebenso wie die Entwicklung eines inzwischen gut eingeführten, partizipativ organisierten Vernetzungskonzeptes von Nutzerinnen/Nutzern und Anbietern technischer Assistenzsysteme und Dienstleistungen.

Ihre eigene weitreichende Vernetzung ist im Zusammenhang dieses Projektes für Kompetenzen im Bereich der interdisziplinären Zusammenarbeit bedeutsam. Ihre Publikationsliste weist 2 Monographien, 6 Artikel/Buchbeiträge in Erst- und 14 in Mitautorenschaft auf, der Schwerpunkt Technik und Alter(n)

findet sich hier wieder.

Fachkompetenz in Bezug auf das Forschungsvorhaben

Durch die personelle Zusammensetzung des Teams ist eine breite Basis an zur Durchführung des Projektes notwendiger Expertise vorhanden; die vorliegenden Arbeiten zeigen hinsichtlich des Forschungsthemas „Menschen mit Demenz‟ sowohl fachliche Kompetenzen, als auch die methodischen Sachkenntnis, den Herausforderungen eines methodenpluralen Designs mit durchaus anspruchsvollen Erhebungs- und Auswertungsmethoden gewachsen zu sein. Von Vorteil für die Durchführung des Projektes und der Dissemination der Ergebnisse ist die bei der Mehrheit vorliegende hervorragende, auf eigener Erfahrung beruhender Kenntnis pflegerischer Berufspraxis. Da einige der Beteiligten bereits früher zusammen gearbeitet haben, steht zu erwarten, dass auch die interdisziplinäre Kommunikation und Zusammenarbeit gelingen.

Scientific relevance, originality and topicality

B 1.1
a) Bedeutsamkeit
Die erfolgreiche Durchführung des hier vorgeschlagenen Projektes würde einen wichtigen Beitrag zur Demenzforschung liefern. Über das Erleben demenziell erkrankter Menschen gibt es inzwischen eine solide Wissensbasis, es verbleiben aber noch viele weiße Flecken. Die betreuungsfreie Zeit ist sicher einer davon, zudem ein methodisch nur schwer zu fassender. Daher ist es berechtigt, hier von einer Forschungslücke und somit von einer zu erwartenden Erweiterung des Kenntnisstandes zu sprechen. Das Design enthält keine neuen Methoden der Erhebung und Auswertung, allerdings ein komplexes Geflecht der Zusammenarbeit mit unterschiedlichen, länderübergreifenden Teams, wodurch forschungspraktische und methodologisch interessante Erkenntnisse zu erwarten sind.

Das Forschungsthema ist sehr gut hergeleitet und argumentativ begründet, was ganz überwiegend auch für die Forschungsfragen und das Forschungsdesign gilt. Geringfügige Einschränkungen bzw. Unklarheiten sind weiter unten angeführt. Insbesondere betrifft das methodologische Fragen und anvisierte, sehr umfangreiche Erkenntnisziele, die z.T. angesprochen, im Design aber nicht expliziert werden (z.B. biographische Fragestellungen oder Fragen unterschiedlicher Kulturen).
Was ist damit gemeint? Welcher Kulturbegriff liegt dem zugrunde? Kulturen, Praktiken und Ordnungen zu beschreiben ist ein äußerst ambitioniertes Programm, das meiner Ansicht nach auch mit dem durchaus sehr guten Projektdesign nicht eingelöst werden kann und auch nicht eingelöst werden muss. Die Kernanliegen, in der dargestellten Weise die forschungs- wie handlungspraktisch bestehende Leerstelle der betreuungsfreien Zeiten zu erhellen, scheint mir Aufgabe genug. Wenn es möglich ist, über die Typologien und die Surveydaten, bei denen die Praktiken im Vordergrund stehen, zusätzliche, auf der theoretischen Ebene liegende Erkenntnisse zu gewinnen, ist das ein erfeulicher Zusatznutzen; die zentrale Argumentationslinie des Antrags zielt jedoch auf den praktischen Nutzen für die alltägliche Pflege von Menschen mit Demenz ab. Das sollte auch so bleiben.
Die korrekte und vom Umfang her angemessene Aufarbeitung des Forschungsstandes ist gegeben.

b) Originalität
Der Ansatz ist originell in zweierlei Hinsicht: Zum Thema selbst ist bislang wenig bekannt, wiewohl es den Alltag einer große, stetig wachsende Gruppe von Menschen betrifft. Originell ist auch die Kombination bekannter und gut eingeführter Methoden und Instrumente zur Erhebung, Auswertung und Synthese der Ergebnisse.

c) Aktualität
Der Alltag und die Lebenssituation der Menschen mit Demenz, ihrer Zugehörigen und Pflegenden sind von steigender Relevanz und bleibender Aktualität, allein durch die hohe Anzahl von der Problematik betroffener Menschen; darüber hinaus ist durch die gegenwärtige Situation des Lebens in einer Pandemie, mit den dadurch nicht zuletzt für diese Personengruppen erschwerten Rahmenbedingungen bis hin zu Besuchsverboten in Langzeitpflegeeinrichtungen, die Aktualität besonders hoch.

Broader impact (forms part of the assessment of scientific relevance, originality and topicality)

B 1.2
Der Forschungsbedarf ist im Antrag umfassend angegeben; auch die jüngst veröffentliche S1-Leitinie der DGP
(Soziale Teilhabe und Lebensqualität in der stationären Altenhilfe unter den Bedingungen der Covid-19 Pandemie) bestätigt deutlich, dass die Frage danach, wie es Menschen mit Demenz ergeht, wenn sie zeitweise auf sich verwiesen sind, eine hohe Bedeutung für ihr Wohlbefinden haben dürfte.
Die Forschungsfragen sind darauf hin angelegt, mindestens in einem zweiten Schritt umsetzungsorientierte Vorschläge zu entwickeln; das Projekt selbst ist stärker grundlagenorientiert, verspricht aber eine gute Basis für umsetzungsorientierte Folgeprojekte zu liefern. Erste Anregungen für die Praxis sind zu erwarten.
Ob unmittelbare Umsetzung in weiteren Bereichen als der Pflege von Menschen mit Demenz und der Verbesserung ihrer Lebensqualität durch Erhellen des 'dunklen Flecks' betreuungsfreier Zeit-Räume zu erwarten sind, hängt sehr von den gewonnenen Erkenntnissen ab. Eine potentielle Weiterentwicklung sprechen die Gesuchstellenden selbst an: die Entwicklung technischer Assistenzsysteme könnte eine potentielle Folge sein.
Die Ausführungen und Argumentationen im Kapitel 2.5 zur wissenschaftlichen und außerwissenschaftlichen Bedeutsamkleit sind überzeugend gelungen; ich teile die Einschätzungen.

Suitability of methods and feasibility

B 2.1
Insgesamt sind die vorgeschlagenen Methoden sehr gut geeignet, das interessierende Feld zu erhellen. Die Gesuchstellenden werden alle Komponenten der Kommunikation mit Menschen mit Demenz einbeziehen, um deren Erleben möglichst gut rekonstruieren zu können. Auch der vorgesehene Einbezug der Pflegenden, um deren Einschätzung sowie Informationen über ihre Handlungspraxis im interessierenden Zusammenhang zu erfahren, isteine wichtige Ergänzung und methodisch sauber angelegt.
Die Bedeutung der Interdisziplinarität wie auch ihre Umsetzung im Projekt sind überzeugend dargestellt. Einige kleinere Kritikpunkte seien dennoch angemerkt:

Eine Präzisierung des erwarteten Nutzens einzelner Komponenten wäre hilfreich. So wären hinsichtlich des Einbezugs zweier Länder, wie bereits weiter oben angemerkt, weitere Argumente zu projektrelevanten Unterschieden und zum Nutzen des Vergleichs der Erkenntnisse wünschenswert. Einige Begriffe bleiben unscharf, etwa "nicht teilnehmende Beobachtung", was ist darunter zu verstehen? Oder "situative Gespräche", S.11, mir ist aus Bewerbungskontexten das Situative Interview bekannt, ist das eine Anlehnung daran?

Die Arbeitspakete sind grundsätzlich plausibel; die Grundstruktur, durch immer wieder gemeinsame Auswertungsschleifen, intensiviert durch insgesamt sechs Workshops, Intersubjektive Nachvollziehbarkeit und größtmögliche Absicherung des Gültigkeitsanspruchs der Ergebnisse zu gewährleisten, ist sehr überzeugend. Die Herausforderung der Einbindung aller Beteiligten bei

gleichzeitiger, nach der jeweiligen Expertise vorgenommener Aufteilung kann so gemeistert werden.
Die Sampling-Strategie sowie die anvisierte Anzahl von Fällen ist sachlich angemessen, das gilt ebenso für die Feldzugänge des qualitativ ausgerichteten Teils und die Gewinnung der Kontakte für die TN des Surveys.

AP 1: excellent gelungen

AP 2. excellent gelungen, Herangehensweise und Umsetzung sind plausibel; interessant wäre noch die anvisierte Dauer der einzelnen Beobachtungen und die Frage danach, wie man sich das ganz forschungspraktisch vorstellen muss: wann sind die Beobachtenden anwesend, wann nicht? Und ist es noch "betreuungsfrei", wenn sie - dann zwar als Einzige - in diesen Zeiträumen anwesend sind, nachdem sie ja den TN als Pflegende oder Betreuende bekannt sind?

AP 3: excellent gelungen, die einzelnen Schritte sind logisch aufeinander aufbauend

AP 4: überwiegend excellent gelungen (s.auch Anmerkungen weiter oben zur Planung der Zusammenarbeit), wünschenswert wären hier noch Details zur geplanten Dissemination, zu der sich nur einige allgemeine Aussagen im Kapitel 2.5.1 finden.

Ein leicht behebbarer Mangel dieses Antrags ist die nicht immer stringente methodologische Fundierung, die (s. Anmerkungen, insbes. im Kapitel 2) an einigen Punkten nicht völlig überzeugend gelungen ist. So wird auf S.11 sehr deutlich, wie die Typenbildung mithilfe der GTM erfolgen soll, weniger offensichtlich ist, welche Erkenntnisse von der Hermeneutischen Sequenzanalyse erwartet werden.
Auch hinsichtlich der Videoanalyse findet sich eine gut nachvollziehbare Begründung für den Einsatz dieser Methode; diese Klarheit würde ich mir für die hermeneutische Sequenzanalyse und die Frage danach, wie (kriteriengestützt? validierend? ergänzend - wenn ja, unter welchem Gesichtspunkt?) der Vergleich von "Beobachtungssituationen und ... Interviewaussagen" (gemeint sind vermutlich die Ergebnisse der jeweiligen Analysen) ebenfalls wünschen.

B 2.2
Die Machbarkeit ist gegeben, Umfang und Dauer der Teilschritte sind, in Relation zur Zusammensetzung und der angegebenen Personenzahl des Projektteams, angemessen.
Auch hier bleiben noch kleine Unklarheiten, die allerdings leicht zu heilen sein dürften:

zum Gliederungspunkt "2.3.6 Zustimmung zur Studienteilnahme" des eingereichten Antrags: Die Belastung der Menschen mit Demenz erscheint mir sehr hoch; es kommen auf sie (a)Teilnehmende Beobachtungen, (b) nichtteilnehmende Beobachtung (unklar, was damit gemeint ist), (c) DCM-Beobachtungen, (d) Videographie (mit unklarer Anwesenheit der Forschenden), (f) wiederholt kognitionspsychologische Verfahren zu; dabei wird (f) zeitlich parallel zu (b) und (c) durchgeführt.

Das geplante Einholen der Ethikvoten halte ich daher für eine unabdingbare Voraussetzung, die im Antrag gegebene Zusicherung, im Falle von nicht selbst einwilligungsfähigen Menschen nach den Maßgaben des „ongoing consent“ zu arbeiten, für essentiell.

Comment

Stärken
•	Die erfolgreiche Durchführung des hier vorgeschlagenen Projektes würde einen wichtigen Beitrag zur Demenzforschung liefern. Über das Erleben demenziell erkrankter Menschen gibt es inzwischen eine solide Wissensbasis, es verbleiben aber noch viele weiße Flecken. Die betreuungsfreie Zeit ist sicher einer davon, zudem ein methodisch nur schwer zu fassender. Daher ist es berechtigt, hier von einer Forschungslücke und somit von einer zu erwartenden Erweiterung des Kenntnisstandes zu sprechen.
•	Das Design enthält keine neuen Methoden der Erhebung und Auswertung, allerdings ein komplexes Geflecht der Zusammenarbeit mit unterschiedlichen, länderübergreifenden Teams, wodurch forschungspraktische und methodologisch interessante Erkenntnisse zu erwarten sind.
•	Das Forschungsthema ist sehr gut hergeleitet und argumentativ begründet, was ganz überwiegend auch für die Forschungsfragen und das Forschungsdesign gilt.
•	Das Erhebungs- und Auswertungsprogramm ist sehr ambitioniert, offensichtlich aber wohl durchdacht.
•	Die Grundstruktur, durch immer wieder gemeinsame Auswertungsschleifen, intensiviert durch insgesamt sechs Workshops, Intersubjektive Nachvollziehbarkeit und größtmögliche Absicherung des Gültigkeitsanspruchs der Ergebnisse zu gewährleisten, ist sehr überzeugend.
•	Die im Antrag geäußerten Einschätzungen hinsichtlich wissenschaftlicher, gesellschaftlicher und lebensweltlicher Relevanz teile ich völlig; entsprechende Veröffentlichungen können eine Bereicherung der jeweiligen Diskurse sein. Der vermutete zukünftige Einbezug technischer Systeme ist grundsätzlich ebenfalls nachvollziehbar begründet, der direkte Zusammenhang zu den zu erwartenden Projektergebnissen allerdings nicht offensichtlich.
•	Durch die personelle Zusammensetzung des Teams ist eine breite Basis an zur Durchführung des Projektes notwendiger Expertise vorhanden; die vorliegenden Arbeiten zeigen hinsichtlich des Forschungsthemas „Menschen mit Demenz" sowohl fachliche Kompetenzen, als auch die methodischen Sachkenntnis, den Herausforderungen eines methodenpluralen Designs mit durchaus anspruchsvollen Erhebungs- und Auswertungsmethoden gewachsen zu sein.

Schwächen
•	Hinsichtlich der Auswertung wären präzise Fragestellungen für die geplanten Methoden hilfreich, nicht zuletzt um die Relevanz und die Aussagekraft der beabsichtigten Zusammenführung besser einschätzen zu können. Für Analysen von TB und Videographie ist das sehr gut, für die geplante Hermeneutische Sequenzanalyse nicht vollständig gelungen.
•	Eine Präzisierung des erwarteten Nutzens einzelner Komponenten wäre hilfreich. So wären hinsichtlich des Einbezugs zweier Länder weitere Argumente zu projektrelevanten Unterschieden und zum Nutzen des Vergleichs der Erkenntnisse wünschenswert. Einige Begriffe bleiben unscharf, etwa "nicht teilnehmende Beobachtung", oder "situative Gespräche". Das gilt auch für theoretische Konzepte wie „Kultur" oder „Ordnungen". Da sie offenbar eine untergeordnete Rolle im Design spielen, war diese Unschärfe für mich nicht entscheidungsrelevant.
•	Das Literaturverzeichnis muss überarbeitet werden, es fehlen zahlreiche Quellen.

Anmerkung
Die Belastung der teilnehmenden Menschen mit Demenz erscheint mir sehr hoch; das geplante Einholen der Ethikvoten halte ich daher für eine unabdingbare Voraussetzung, die im Antrag gegebene Zusicherung, im Falle von nicht selbst einwilligungsfähigen Menschen nach den Maßgaben des „ongoing consent" zu arbeiten, für essentiell.

Note on the evaluation procedure

The evaluation bodies of the SNSF strive to reach a balanced overall assessment of each proposal. External reviews play an important role in this. Reviewers generally review only one proposal. The evaluation bodies of the SNSF, however, must compare and rate the quality of all proposals submitted by a given deadline. The opinions expressed in external reviews are generally positive, or they may occasionally include critical remarks that are largely irrelevant to the assessment conducted by the evaluation body. Therefore, the final decision taken by the SNSF evaluation bodies need not necessarily reflect the content of external reviews.
